# The Estimate of Parental Quality of Life Loss Due to Respiratory Syncytial Virus (RSV) Hospitalization

**DOI:** 10.3390/diseases11040126

**Published:** 2023-09-24

**Authors:** August Wrotek, Oliwia Wrotek, Teresa Jackowska

**Affiliations:** 1 Department of Pediatrics, Centre of Postgraduate Medical Education, Marymoncka 99/103, 01-813 Warsaw, Poland; tjackowska@cmkp.edu.pl; 2 Department of Pediatrics, Bielanski Hospital, Cegłowska 80, 01-809 Warsaw, Poland; 3 Student Research Group, Bielanski Hospital, Cegłowska 80, 01-809 Warsaw, Poland

**Keywords:** respiratory syncytial virus, bronchiolitis, pneumonia, caregiver, infant, quality-adjusted life year, health-related quality of life, EQ-5D, cost effectiveness

## Abstract

Background: Respiratory syncytial virus (RSV) is one of the leading causes of pediatric hospitalizations, mainly in children under 2 years of age. Hospitalization affects the caregivers’ quality of life (QoL). We assessed the caregivers’ QoL during RSV-confirmed hospitalizations of children under 2 years old, identified the most affected QoL dimensions and calculated utilities focusing on the assessment methods and potential confounders. Methods: The caregivers filled out the EQ-5D questionnaire, consisting of a descriptive system (assessing 5 QoL dimensions) and a visual analog scale (EQ VAS). Utility, utility loss and quality-adjusted life years (QALY) loss were calculated, and a concordance between the two systems was assessed. Results: A disturbance in any of the five assessed dimensions was reported by 42% (55 out of 132) of the caregivers, mostly anxiety/depression (37%) and pain/discomfort (17%). The utilities varied between 0.17 and 1 in the descriptive system and 0.33–1 (median 0.86) in the EQ VAS, with a utility loss of 0.14 (IQR: 0.1–0.2). The calculated QALY loss reached a median of 2.45 × 10^−3^ (IQR: 1.37 × 10^−3^–4.56 × 10^−3^) and was not influenced by the patient’s age or the final clinical diagnosis (QALY loss for bronchiolitis: 2.74 × 10^−3^, pneumonia: 1.84 × 10^−3^, bronchitis: 1.78 × 10^−3^, differences statistically insignificant). Only a moderate concordance between the descriptive system and the EQ VAS was seen (Spearman’s rank correlation coefficient = 0.437, *p* < 0.05), with the latter revealing a higher degree of QoL disturbances. Conclusions: RSV hospitalization influences parental QoL significantly, and anxiety/depression is the most commonly reported issue. Utility impairment scores depend on the assessment method but not on the patient’s age or final diagnosis. Thus, the impact of RSV on caregivers’ QoL cannot be underestimated.

## 1. Introduction

Human respiratory syncytial virus (RSV) is one of the leading etiological factors of acute lower respiratory tract infections (ALRI), holding responsibility for approximately 25.4–44.6 million cases a year [1]. A huge number of cases lead to hospitalization, and in a global context it is estimated that around 2.9–4.6 million hospital admissions take place annually; the vast majority of hospitalizations take place during the first 2 years of life [1,2,3,4]. While the disease course may vary hugely from a transient episode, through an outpatient ALRI to an in-patient treatment, the risk of complications is high, with a predominance of acute otitis media (risk up to ca. 50%) and pneumonia (up to 33%), although the latter is often treated rather as a disease presentation than a sequela [5,6,7,8]. A typical presentation of the RSV infection is bronchiolitis, of which the RSV is responsible for around 70% of the cases; bronchiolitis is diagnosed in children under 2 years of age, and an intensive care unit (ICU) transfer is needed in 2–6% of the cases, although some estimates suggest up to 15–20% of the patients temporarily require treatment at the ICU; in some of the cases, mechanical ventilation may be needed [9,10]. In Poland, the rates of RSV hospitalization increased significantly in recent years and in the past decade reached a mean of 267.5/100,000 children and 1132.1/100,000 infants a year; the tendency towards an increasing number of hospitalizations was also observed in other European countries and may be due to an increased RSV circulation and/or virulence, but it also may be related with the improvement of diagnostic methods, including higher availability as well as greater RSV awareness among health care professionals [4,11].

The general high percentage of the cases requiring hospital treatment results from both the RSV virulence and the age of the affected patients. On one hand, the RSV induces various pathomechanisms, including the impairment of the mitochondrial respiratory function, changes in the cell metabolism (by facilitating glycolysis) and a generally attenuated immune response, which result in an increased viral replication; on the other hand, an inadequately enhanced immune response may result in airway hyperresponsiveness; these interactions may be intensified depending on the individual patient’s response or environmental factors and may further decide on the clinical presentation and the disease severity [4,12,13]. In Poland, the most frequent RSV-related diagnoses among hospitalized children are pneumonia (53% of the cases), followed by bronchiolitis (33%) and bronchitis (18%) [4].

The quality of life (QoL) during an RSV infection may be hugely affected in children due to dyspnea, increased respiratory effort, cough, fever or impaired feeding, and this state may last for a period of time [14,15,16,17]. Hospitalization of a child is related to numerous diagnostic and treatment procedures and generates high levels of stress, and up to more than one-quarter of parents experience post-traumatic stress symptoms after a hospital discharge [18,19,20]. Factors that increase the risk of a higher level of stress include the child’s health state, length of hospital stay and age, but they also include one’s former experiences with hospital treatment, educational level, individual stress reactions or personal situations [18,19,21,22]. Thus, aside from the patients’ challenged QoL, parents/caregivers whose child is affected by the disease may themselves reveal a decrease in their own QoL; in the literature, a respiratory hospitalization of a preterm infant was related to a higher caregiver overload, or lower values in the physical QoL assessment questionnaires [23]. 

The parents of children hospitalized due to bronchiolitis mostly experience guilt and anxiety, and the need to take an active role in the child’s treatment often remains unmet [24]. The hitherto conducted studies mainly focused on premature infants, although the majority of the RSV cases take place in otherwise healthy children; the published data on the topic of RSV-related QoL loss are scarce with regard to pediatric patients, and an even larger gap exists in terms of the caregivers’ QoL. A systematic review by Glaser assessed the influence of the RSV on the caregivers’ QoL, showing a significant reduction in both caregivers’ and family’s QoL [16]. Similarly, the problem of the scarcity of QoL assessment affects many childhood illnesses and also patients with chronic diseases, where more attention should be paid to the emotional domain of QoL, considering positive factors that could improve patients’ well-being. [25,26]. 

Although numerous methods have been created and evaluated for the purposes of the measurement of QoL, it is a challenging task; the most commonly used methods can be divided into direct and indirect assessment [27]. The first group of methods implies a direct question on the QoL under different health conditions and includes the visual analog scale (VAS), standard gamble or time trade-off scale; the indirect methods are based on questionnaires filled out by patients, and the results are then translated into utilities with the use of locally verified value sets [27]. EQ-5D was first introduced in 1990 (in Poland, it was translated for the first time in 1997) and combines two methods of assessment: a descriptive system and the EQ visual analog scale (EQ VAS) [28,29]. The descriptive system addresses five dimensions (mobility, self-care, usual activities, pain/discomfort and anxiety/depression), each with three levels, while the EQ VAS is a 100-point verticular scale [28]. The EQ-5D generates results by two different methods: the EQ VAS returns a numerical value, whereas the responses to the EQ-5D are converted further into a number based upon locally (country or region) established value sets; the value sets may be calculated with the use of time trade-off, VAS or discrete choice experiments [28]. The Quality Adjusted Life Year (QALY) is a globally used pharmacoeconomic metric that allows an assessment of healthcare technologies and facilitates comparisons between various diseases and populations; its calculation is based upon multiplying the utility variations (decrease due to a disease or increase after an intervention) by time in order to quantify the QoL [27]. 

A measurement of health utilities is also necessary for cost-effectiveness analyses, and significant development in preventive strategies may currently be observed, including monoclonal antibodies and novel vaccine candidates; thus, with the growing number of prophylactic measures as well as possible therapeutic strategies, a detailed assessment will require data on the benefits, and the benefits might reach out far beyond the sole protection against the disease [30,31,32,33]. The rationale underlying this study is the identification and evaluation of parental QoL challenges related to RSV hospitalization. In this study, we focused on the age group most affected by RSV, i.e., children under 2 years of age, thus only caregivers of the youngest patients were eligible. We aimed to identify the dimensions of QoL most affected, calculate caregiver utilities during hospitalization, convert them into QALYs and assess the concordance between direct and indirect QoL assessment methods and the influence of potential confounders, like a child’s age or final diagnosis.

## 2. Materials and Methods

### 2.1. Conceptual Framework—Study Objectives, Research Hypotheses and Expected Results

Due to the scarcity of data on this topic, in this study we first sought to identify the dimensions of caregivers’ QoL affected by hospitalization of children with the RSV disease. Detailed objectives included identifying the frequency and level of impairment of 5 QoL dimensions. Another major objective was to estimate the reduction in caregivers’ utility due to a child’s RSV hospitalization and the loss of QALYs; to make the calculations as precise as possible, we used both the descriptive system and the visual analog scale and compared concordance between them. Finally, we aimed to assess the influence of potentially important factors: the child’s age, final diagnosis and length of hospital stay. 

We hypothesized that (1) the most commonly affected area would be anxiety/depression or pain/discomfort, (2) QoL impairment would not be marginal, affecting up to 50% of caregivers, while (3) the severity of impairment would be mild to moderate in the vast majority of cases. In terms of utility, we expected our results to be in line with those previously published but hypothesized that there might be significant differences between the systems used for assessment. We also assumed that none of the potential confounders (child’s age, final diagnosis, length of hospital stay) would significantly alter parental QoL. 

### 2.2. Selection Criteria

We performed a prospective survey study on caregivers of children under 2 years of age who were hospitalized because of a laboratory-confirmed RSV lower respiratory tract infection. This was part of a QoL project that aimed to assess the QoL of both children and caregivers during hospitalization due to the most frequent conditions in the Pediatric Ward of the Bielanski Hospital, Warsaw. 

The major inclusion criterion was being a caregiver (preferably a parent or legal guardian) of a <24-month-old child hospitalized due to a community-acquired RSV infection. The caregivers of those children were invited to take part in the study, and after their informed consent was obtained, they were enrolled in the study. We excluded caregivers who did not fill out the questionnaires, did not respond to the follow-up (a phone contact at least one week after a hospital discharge) or transferred to another hospital. In the case of more than one caregiver attending a child, the study protocol allowed both caregivers to complete separate questionnaires, while the caregivers who were not attending the children every day were excluded. If the caregivers switched in custody, the one who was spending more time at the hospital was encouraged to fill out the questionnaire (if the informed consent had been obtained). With regard to the legal status of the caregiver, in order to make the study group as homogenous as possible, we included only those caregivers who were legal guardians of the child. 

We previously published detailed information on the inclusion of pediatric patients into the QoL assessment [17]; the following definitions were used:

- symptomatic patient: a child under 24 months of age who presented with at least one of the following signs/symptoms: coryza, cough, increased body temperature or dyspnea;
- laboratory confirmation of an RSV infection: a positive rapid antigen test or RT-PCR result; a nasopharyngeal swab taken at the hospital admission and Alere BinaxNOW (Alere Scarborough Inc.; Scarborough, ME, USA) or RSV Xpert Xpress Flu/RSV XC GeneXpert (Cepheid, Sunnyvale, CA, USA), respectively, performed according to the manufacturer’s instruction;
- community origin of the infection: signs/symptoms of the respiratory tract infection commenced before the hospitalization or up to 48 h after the admission;
- final diagnosis (International Classification of Diseases, 10th Revision, ICD-10 are showed in brackets): pneumonia (J12.1), bronchitis (J20.5), bronchiolitis (J21.0). The detailed diagnostic criteria have been published elsewhere [17];
- additionally, in the case of pneumonia, the final diagnosis was confirmed with an imaging study (abnormalities on chest X-ray or lung ultrasound consistent with pneumonia) [17].

Detailed inclusion and exclusion criteria are summarized in Table 1. 

### 2.3. Study Group 

**Figure 1:**
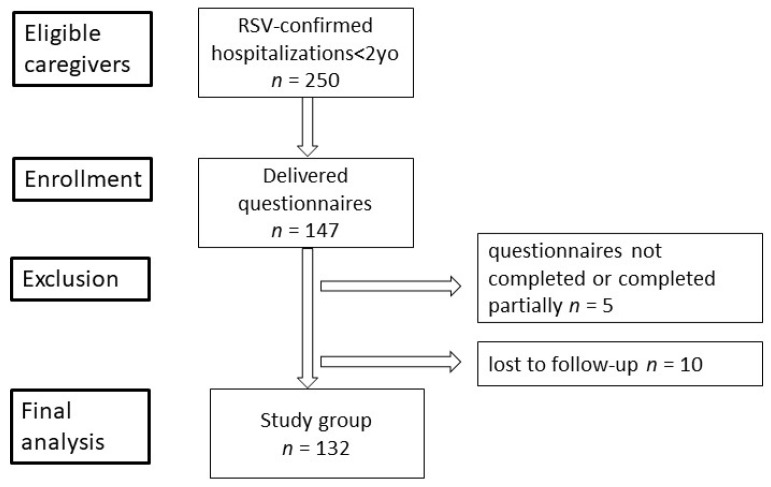
A flowchart of the study participants.

During the study period, there were 250 laboratory-confirmed RSV hospitalizations, and 147 caregivers were offered participation in the study. The informed consent was obtained from the caregivers, all of whom were legal guardians to the patients as well. Five questionnaires were completed inappropriately (i.e., not fully or not completed at all), and no cases of discharge on parental request or another hospital transfer took place, but in 10 cases the follow-up assessment failed; thus, those patients were excluded from the study (Figure 1). 

The caregivers were taking care of 132 patients, aged 10 days to 720 days (median 3.8 months, interquartile range, IQR: 2.05–6.88 months), whose length of stay varied between 3 and 16 days (median 7 days). The children were diagnosed with bronchiolitis in 100 cases, pneumonia in 22 cases and bronchitis in 10 cases (the detailed baseline characteristics of the children are shown in Table 2).

### 2.4. Study Procedures

The children: 

The children whose caregivers participated in the study did not undergo any additional procedures as a result of the study. 

The caregivers: 

The caregivers were asked to fill out the EQ-5D-3L questionnaire at the end of the hospitalization. 

Instruments

EQ-5D: concept, valuation and use

The EQ-5D-3L was introduced in 1990 with the purpose of providing a short easily comprehensible questionnaire, which would provide a generic measure of health status and could therefore be used in clinical trials, observational studies, population health surveys and longitudinal studies to assess health status changes over time [28]. The EQ-5D-3L is designed for self-completion by respondents, and we used the paper mode of administration. In the 3-level version of the EQ-5D, a patient (caregiver in this case) answers two parts of the questionnaire: the descriptive part and the EQ VAS. As shown above, the descriptive system refers to 5 different dimensions, and for each of them, the patient ticks one of three boxes that correspond to one of three levels: no problems, some problems and extreme problems. Sample questions in the English version include the following sentences (to be ticked) on the pain/discomfort dimension:—I have no pain or discomfort, —I have moderate pain or discomfort, —I have extreme pain or discomfort, or on the anxiety/depression dimension:—I am not anxious or depressed, —I am moderately anxious or depressed, —I am extremely anxious or depressed changes [28]. The level ticked by the patient in the questionnaire is expressed by a 1-digit number, and the answers regarding the five dimensions create a 5-digit combination that the describes patient’s health state with 243 (3^5^) possible health states. Then, to quantify the QoL, each of the health states is converted into a numerical value with the use of value sets, which are estimated in the studies using the time trade-off (TTO) valuation technique or the visual analog scale (VAS) valuation technique or both. Value sets are generated for specific populations and numerous sets are available for different country societies from 6 continents [28]. Local preferences may vary between and within countries and may be influenced by age, gender, standard of living, health expenditure or unemployment [34,35]. For countries without regional scores, estimated regional population norms can be used; an algorithm has also been developed to map responses from the EQ-5D-3L to the scores of the newer, more sensitive 5-level questionnaire (EQ-5D-5L) [35,36]. The EQ-5D-5L questionnaire is characterized by improved measurement properties compared to the 3L (in terms of lower ceiling effect, better informativity) with an upward shift in utility due to less severe (although more frequent) reports of QoL disturbances; however, the superior measurement properties were more pronounced in the population with multiple morbidities [37]. Here we used the Polish value set based on the study by Golicki, who used the time trade-off method and only the EQ-5D-3L value sets were available at the protocol designing stage and at the study commencement [29]. During the same QoL evaluation, the patient is asked to use a 100-point VAS and indicate the health status (where “0” means “best imaginable health state”, while “100” is labeled as “the worst imaginable health state”). 

Follow-up:

Caregivers who completed the questionnaire were contacted at least 1 week after hospital discharge and asked to rate their current QoL using a 0–100 point scale. The result was included in the QALY calculations as a kappa coefficient. 

Instruments’ application timing

The EQ-5D questionnaire asks participants to report on their QoL on the day it is completed. The patient’s condition during hospitalization for RSV is dynamic and may change on a daily or even hourly basis, and the patient’s condition may subsequently also influence the caregiver’s QoL. However, for ethical reasons, we decided to assess QoL once at the end of the hospitalization in order to allow caregivers to focus on the children they were caring for without unnecessary distractions. To check QoL during hospitalization and in the disease-free period, we conducted a follow-up. The aim of this follow-up was to eliminate the potential influence of any chronic condition that might affect the caregivers’ QoL, so all caregivers who reported any signs/symptoms of a disease (other than the chronic condition present at the time of hospitalization) were contacted again in a disease-free period.

### 2.5. Study Endpoints

The primary endpoints consisted of the following: (1) a recognition of the QoL dimensions that are affected most often, (2) a calculation of degree of disturbances in the affected dimensions, (3) a calculation of parental utility during the RSV hospitalization and (4) an assessment of the QALY loss, while the secondary objective was to compare the degree of concordance between the descriptive system and the EQ VAS in the case of RSV hospitalization and its dependence on the length of stay. The analysis of the QALY was performed in subgroups based upon the final ICD-10 diagnosis and with regard to the patients’ age subgroups. For the latter, the patients were assigned to 4 age groups: <3 months old, 3–5 months old, 6–11 months old, 12–23 months old, and a separate month-by-month analysis was also performed. The age limits were set in the above manner based on the risk groups for a severe course of bronchiolitis (<3 months old) and pneumonia (<6 months old) in the Polish guidelines on respiratory tract infection treatment [38]. 

### 2.6. Utility and QALY Calculations

As shown above, the utilities in the EQ-5D were assessed with two different methods. 

First, the utilities received from the descriptive system and from the EQ VAS were computed; since each questionnaire was completed once at the end of the hospitalization, we assumed that it reflected the whole period of the in-hospital treatment. The utilities were calculated in 4 different steps:

(1) The utility during hospitalization was calculated by assigning the received values to each day and the total utility was computed by multiplying each of the results (descriptive system and EQ VAS) by the length of stay.
(2) The utility outside the hospitalization was estimated by multiplying the baseline utility (a kappa coefficient, obtained on the follow-up in a disease-free period) by the number of days outside this hospitalization (i.e., 365-length of stay).
(3) The expected utility (if hospitalization had not taken place) was calculated by multiplying 365 by the baseline kappa coefficient.
(4) The utility loss was assessed by subtracting the sum of the utilities (1 + 2) in the year with hospitalization from the theoretical value of the expected utility that would not be disturbed by the in-hospital treatment (3).

The QALY loss was obtained by dividing the above utility loss (4) by the expected utility (3). 

### 2.7. Statistical Analysis

The data distribution was verified with the Shapiro–Wilk test and the data are presented as a mean +/− standard deviation (in the case of normally distributed data) or median with interquartile range (if the distribution was not normal). Comparisons of the groups were executed with corresponding parametric tests (Student *t* test to compare two groups or ANOVA with Bonferroni correction to compare multiple groups) or nonparametric tests (Mann–Whitney U test or Kruskal–Wallis test with multiple rank comparison, respectively), and Wilcoxon matched-pairs test for the comparison of the QoL assessment results with different assessment methods. To test if the QoL dimensions (in the descriptive system) are influenced by the patient’s age, the two-way Chi-squared test was carried out. Spearman’s rank correlation test was performed to compare the QoL results from the descriptive part of the questionnaire with the EQ VAS and the correlation between the length of stay and utility/utility loss in both methods (no assessment of the correlation with the QALY loss was performed, since the QALY calculations formula included the length of stay). The limit of statistical significance (a *p*-value) was set below 0.05. The statistical analysis was performed with Statistica 13.1 software (Statsoft, Tulsa, OK, USA). 

The study was approved by the local Ethics Committee at the Centre of Postgraduate Medical Education, Warsaw with the permission (number 115/PB/2018) issued on 7 November 2018). The study was conducted in accordance with the Declaration of Helsinki with its later amendments. An informed consent was received from a patient prior to the study enrollment.

## 3. Results

The study group consisted of 132 members and the median baseline QoL (kappa coefficient) was 1.0 with the responses to the question on the baseline QoL varying between 0.9 and 1 (Table 3).

**Figure 2:**
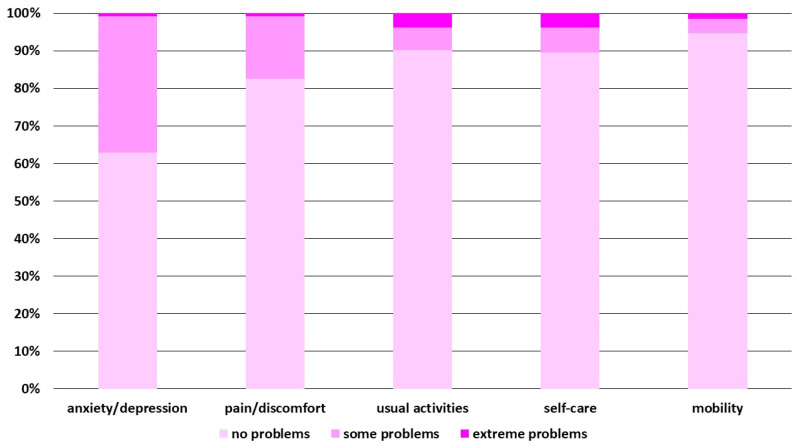
Influence of RSV hospitalization on various dimensions of the caregivers’ quality of life. The graphs show a percentage share of 3 levels of the answers to each of the questions: no problems, some problems, extreme problems; the graphs are based on the responses from the EQ-5D descriptive system.

A disturbance in at least one of the five assessed dimensions was reported by 42% of the caregivers, with a predominance of the anxiety/depression dimension (37%). Within this area, some problems were reported by 36% of the caregivers (*n* = 48), while extreme problems by 0.8% (*n* = 1). The second most affected dimension was pain/discomfort (17% of the respondents) with some problems reported in 17% of the cases (*n* = 22) and extreme problems in 0.8% (*n* = 1). The remaining dimensions were affected to a minor degree, with problems within usual activities reported by 10% of the caregivers, self-care in 8% and mobility in 5%. Detailed frequencies of the answers are shown in Figure 2. 

The EQ-5D descriptive system responses were converted into numerical values with the use of the Polish time trade-off values [29]. The reported utilities varied between 0.17 and 1, with a median of 1. The EQ VAS answers showed utilities between 0.33 and 1, with a median utility of 0.86 (IQR: 0.8-0.9); corresponding utility losses reached a median of 0 (IQR: 0–0.106) in the descriptive system and 0.14 (IQR: 0.1–0.2) in the EQ VAS (Table 4). 

**Figure 3:**
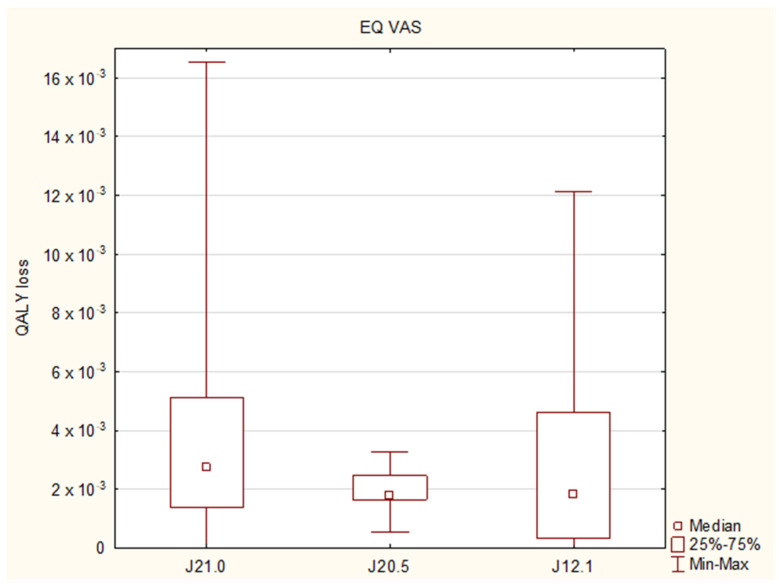
Quality-adjusted life years (QALY) losses with regard to the final diagnoses: J21.0 RSV bronchiolitis, J20.5 RSV bronchitis, J12.1 RSV pneumonia. The results from the EQ VAS assessment.

**Figure 4:**
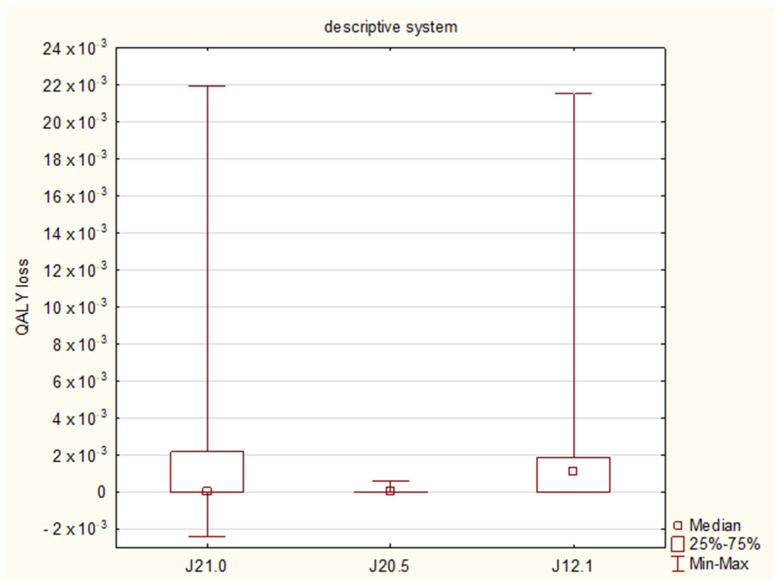
Quality-adjusted life years (QALY) losses with regard to the final diagnoses: J21.0 RSV bronchiolitis, J20.5 RSV bronchitis, J12.1 RSV pneumonia. The results from the descriptive system assessment.

The calculated QALY loss reached a median of 0 (IQR: 0–1.83 × 10^−3^) and 2.45 × 10^−3^ (IQR: 1.37 × 10^−3^–4.56 × 10^−3^), when QALY was evaluated with the use of the descriptive system and the EQ VAS, respectively (Table 4 and Table 5). With regard to the final diagnoses, the EQ VAS revealed that bronchiolitis affected the QALY the most (QALY loss 2.74 × 10^−3^), followed by pneumonia (1.84 × 10^−3^) and bronchitis (1.78 × 10^−3^) (Figure 3), while the descriptive system showed the median values of 0, 1.13 × 10^−3^ and 0, respectively (Figure 4); the Kruskal–Wallis test showed no significant differences between the final diagnosis-based groups in either of the scoring systems (*p* = 0.1799 and 0.0724, respectively).

**Figure 5:**
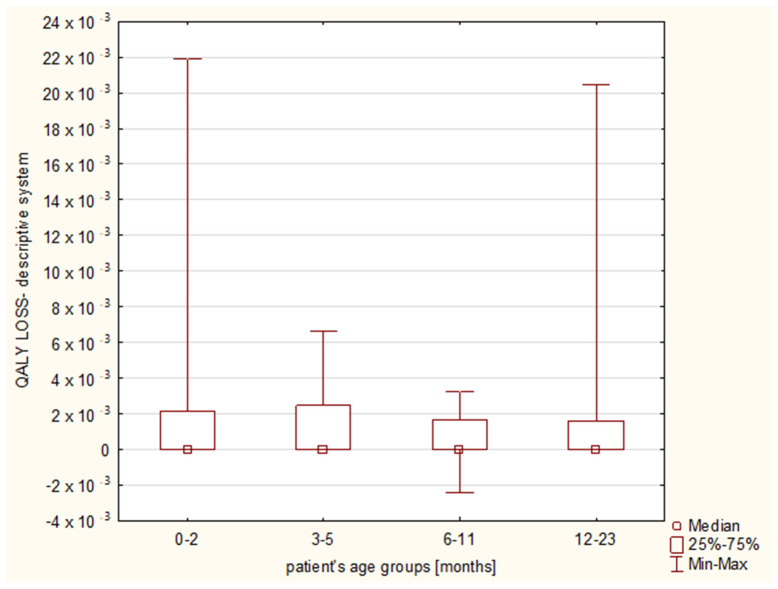
Quality-adjusted life years (QALY) losses with regard to the age groups (0–2 months, 3–5 months, 6–11 months and 12–23 months old). The results from the descriptive system assessment.

**Figure 6:**
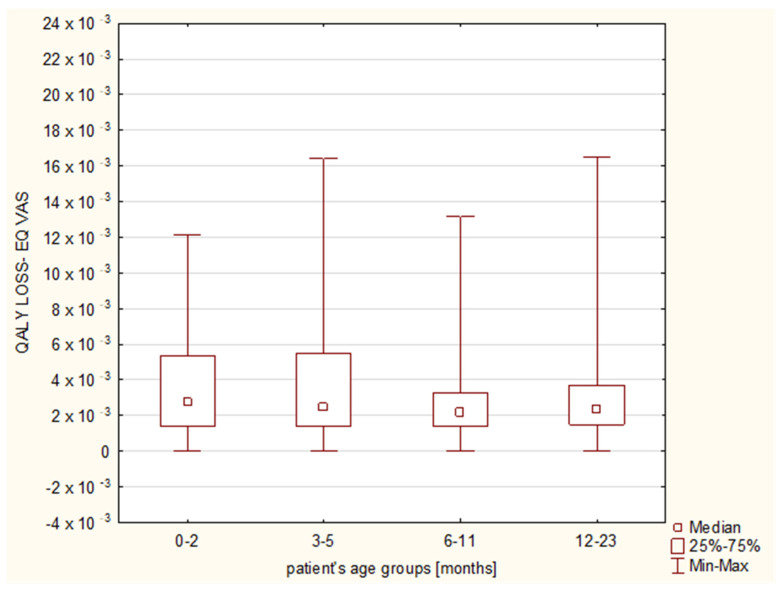
Quality-adjusted life years (QALY) losses with regard to the age groups (0–2 months, 3–5 months, 6–11 months and 12–23 months old). The results from the EQ VAS assessment.

The patients’ age groups analysis as well as the month-by-month age analysis showed no statistically significant differences regarding the utility, utility loss or the QALY loss in any of the assessment tools (Figure 5 and Figure 6). No influence of the patient’s age group on more frequent disturbances among particular QoL dimensions was observed in the descriptive system. 

**Figure 7:**
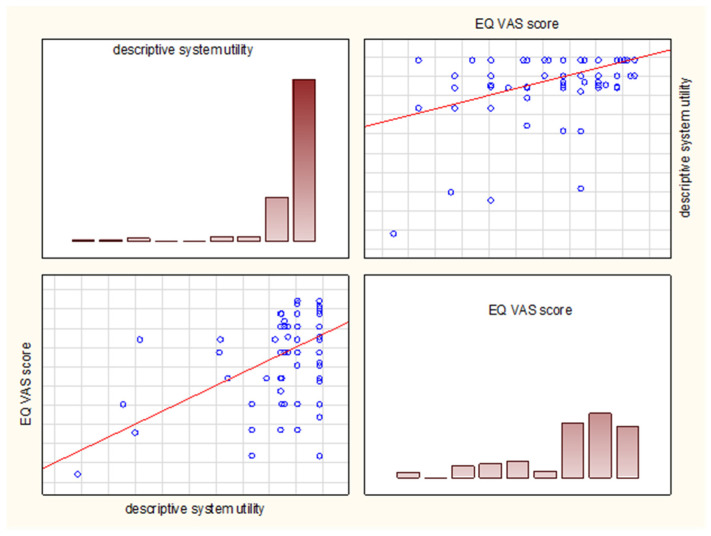
The correlation graphs of the utility assessment between the descriptive system and EQ VAS. The results are based on the Spearman’s rank correlation test.

We observed a moderate concordance between the descriptive system and the EQ VAS, and the Spearman’s rank correlation coefficient reached 0.437 (*p* < 0.05) (Figure 7), while the subgroup analysis showed the only statistically significant correlation coefficient (rho = 0.462, *p* < 0.05) in the bronchiolitis (J21.0) subgroup without statistically significant correlations in the pneumonia and bronchitis subgroups. 

**Figure 8:**
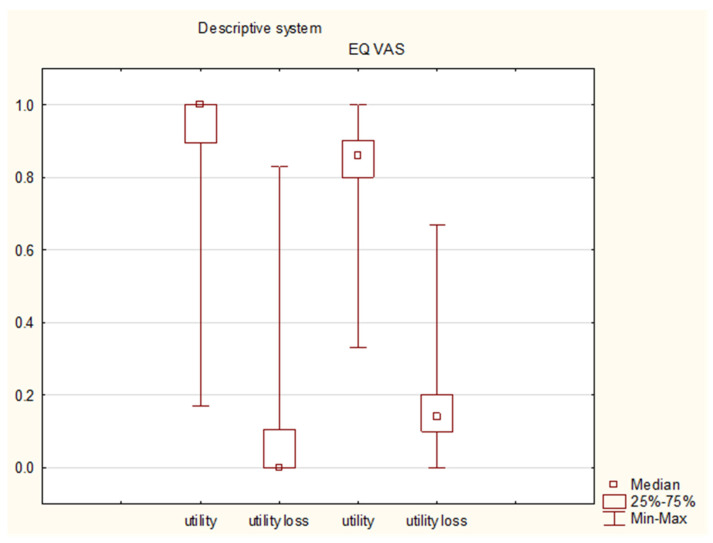
A comparison of the caregivers’ utilities and corresponding utility losses in the descriptive system (on the left) and the EQ VAS assessment (on the right).

A higher degree of QoL disturbances was seen in the EQ VAS assessment (*p* < 0.01 for the utility, utility loss and QALY loss in the Wilcoxon matched-pairs test) (Figure 8). 

Of note, we observed a mild correlation between the length of stay and the utility (as well as a corresponding utility loss) in the descriptive system but not in the EQ VAS assessment.

## 4. Discussion

The influence of the RSV on quality of life is gaining more attention and is becoming an important issue, since on the one hand, QoL is disturbed significantly, and on the other hand, the development in the field of prophylaxis will require cost-effectiveness analyses that include an assessment of QoL [14,16,17,39,40]. Nonetheless, the evidence on the RSV-related deterioration in QoL is scarce with regard to pediatric patients, and it is even scarcer regarding the QoL of the caregivers. Our study shows that the RSV affects various aspects of caregivers’ lives, resulting in a significant deterioration of its quality. 

The existing research recognizes the high stress levels experienced by the parents of RSV-hospitalized infants, and, as expected, the results of our study also exhibit the effects of parental stress [41]. We observed that the anxiety/depression dimension was affected most frequently among the caregivers, which is in line with previously published studies [39,40]. The analysis performed by Leidy et al. in a group of 46 children under 30 months of age (with a history of prematurity—the study included children born ≤35 weeks of gestational age) found a high level of anxiety and stress, combined with poorer general health compared with controls [39]. A systematic review by Gates focused on bronchiolitis studies, since bronchiolitis is a typical clinical presentation of the RSV infection, and disclosed that the parents of the hospitalized children experienced guilt and anxiety [24]. Certainly, anxiety is an important stress factor, and both anxiety and stress influence overall health, which, when affected, may in turn intensify the perception of stress and anxiety, and a holistic approach might help elucidate the combined effects; however, attempts to divide the particular dimensions should be made in order to facilitate understanding of the impact of the RSV disease and prompt targeted counteractions. High levels of stress were also observed by Pokrzywinski and colleagues, who analyzed hospitalizations of preterm infants born at 29–35 weeks of gestational age and, interestingly, the study found that the caregivers’ stress even exceeded infants’ stress perceived by caregivers [40]. The study by Pokrzywinski et al. seems crucial for understanding the potential severity of the RSV impact on the caregivers, since the analysis showed that the caregivers’ stress has a higher magnitude than the infant’s stress (as perceived by the caregivers) [40]. Several areas of interest have been proposed to decrease parental distress, including decreasing the risks of readmission or reinfections and painful procedures, positive experiences with healthcare professionals and their awareness of the latest developments; offering the caregivers a more active role in taking care of the child may also mitigate their anxiety; a “client-oriented” approach would promote patient well-being as the primary goal of health care [24,42,43].

With the exception of the stress magnitude during hospitalization, noteworthy is the fact that 42% of the patients experienced disturbances in at least one of the dimensions, albeit the second most affected dimension (pain/discomfort) was present in a much lower percentage of caregivers than anxiety/depression (17% vs. 42%). Furthermore, the level of the disturbances was in the majority of cases moderate, which supports the thesis that the descriptive system does not have to reflect the influence of RSV hospitalization on caregivers in terms of the severity of the QoL disturbances as precisely as possible. Although it has been shown that EQ-5D reveals the lowest scores in acute childhood infections, while other tools (like the Health Utilities Index Mark 2, HUI2) reflect lower values in chronic conditions [44], it needs to be remembered that the EQ-5D questionnaire is a health status assessment tool, and as such it does point out the caregivers’ health areas that are affected the most. Nevertheless, the discordance between the descriptive system and the visual analog scale needs to be explained. Firstly, the descriptive system is based on five dimensions, among which only the anxiety/depression dimension might have been expected to reveal significant disturbances, while the EQ VAS is a more intuitive self-assessment tool that gives a general answer to the question of the respondent’s health state. Thus, health states indicated by the descriptive system in the case of a child’s RSV hospitalization show a less severe utility loss than the EQ VAS, and the correlation between these two assessment systems is reflected in this issue with only a moderate correlation (the correlation coefficient reached 0.437). The single most striking observation to emerge from the assessment methods’ comparison is a higher degree of QoL disturbances observed when the EQ VAS was applied; however, in order not to overestimate the visual analog scale, we propose that the systems should rather be treated as complementary methods in this case; while the descriptive system underscores the health areas that are affected the most, the EQ VAS returns numerical values on true utility loss. 

The QALY loss calculated from the EQ VAS revealed a median value of 2.45 × 10^−3^, which is a slightly lower QALY disturbance than the one computed by Glaser et al. in the systematic review that included children under 5 years of age, aimed to determine the utility and QALY losses in both children and their caregivers [16]. The net QALY loss presented in the review reached 3.1 × 10^−3^, and the value was adjusted for prematurity, since the original studies included preterm infants [16]. It needs to be underlined that although the previous studies focused on preterm infants and their caregivers, the majority of the RSV cases are observed mostly in otherwise healthy children; thus, the protocol of our study assumed the inclusion of a cohort of children hospitalized in a regular pediatric ward, and the use of the baseline health status assessment (kappa coefficient) practically eliminates the influence of other health disorders. Interestingly, in a few cases, the utilities (and the corresponding QALY losses) calculated in the descriptive system exhibited negative values, which would imply an improvement in the caregivers’ utility due to the child’s hospitalization—this paradoxical phenomenon is easily explicable by the utility calculation formula, which uses value sets; a combination of five answers is assigned to the health status determined in the population study, and, as seen in our study, a particular set of answers in the population study might have been related to a better health status than the self-assessment by the patient in the follow-up [29]. Except for this minor contradiction, a separate general comment on the value sets is needed as well; the value sets are calculated for particular populations, mostly countries, and the results obtained from the (actually) same answers to the questions on the five dimensions may vary. The differences may derive from the methodological aspects of the value sets calculations [45] but may also be related to the cultural, linguistic, urban/rural residence, healthcare system or sociodemographic differences; while the regional value sets are preferred, in the case of lack of local estimations, supra-national value sets for clusters of countries might be used in order to optimize the assessment [46,47,48]. Regarding our study, we need to emphasize that the local Polish value sets were used for the descriptive system, while the EQ VAS seems to be a more universal assessment. 

Interestingly, the EQ VAS results, in contrast to the descriptive part, were free from the influence of the length of stay—there was no correlation between the duration of hospital stay and the EQ VAS score; we can only hypothesize that when the hospitalization period is longer, an increased impact on the other health areas gains importance, for example, one’s usual activities, self-care or even mobility. 

The QALY losses did not depend on the child’s age or the final ICD-10 diagnosis. This is a significant finding, since the relationship between the age and the QALY scores might have been expected due to a generally higher severity of the disease in younger children; although the differences in the patients’ ages were relatively narrow in our research group (24 months), in epidemiological studies it has been observed that the vast majority of the RSV hospitalizations take place in the first year of life, and the age-based classifications have shown that the youngest infants are running an especially high risk of hospitalization and/or a severe disease course [1,2]. In the context of QoL, a study by Hodgson observed the differences between younger and older RSV patients (i.e., under and over 5 years of age) [14], while we observed no age-attributable differences among children under 2 years old [17]. Nor did the final diagnosis (bronchiolitis versus pneumonia versus bronchitis) influence the variations in the caregivers’ utility and QALY loss. In the descriptive system, the patient’s age was not related to the specific health dimension disturbances. Taken together, these findings emphasize the huge impact of RSV hospitalization on the caregivers’ QoL, regardless of the child’s age or diagnosis. 

It needs to be underlined that the impact of RSV hospitalization reaches far beyond a transient (during the hospital stay) deterioration of QoL, resulting in work absenteeism or generating a lot of out-of-pocket expenses; also, the time horizon of the RSV hospitalization effects remains unknown; Leidy et al. observed significant distress among caregivers, mostly anxiety, up to 60 days post-discharge, while our study protocol assumed an in-hospital QoL assessment only [39,41].

There are certain strengths and limitations to this study. To the best of our knowledge, this is one of the very few analyses of the caregivers’ QoL related to RSV hospitalization. However, the generalizability of our results is subject to certain limitations. Firstly, it was a single-site study, which alongside the relatively small sample size does not allow us to make generalizations with a high level of certainty. Secondly, the results based upon a subjective assessment are susceptible to cause bias and limited by the methods themselves (the descriptive system and the visual analog scale), although the use of two different methods was expected to contribute to a deeper understanding of the caregivers’ QoL during RSV hospitalization. It should be emphasized that the EQ-5D questionnaire was completed once by each participant at the end of hospitalization, so it reflects their QoL at the end of hospitalization rather than when the patient was at their worst. In addition, the time horizon, which assumes only the assessment of the hospitalization period, does not assess the loss of QoL after discharge; on the other hand, it reduces the risk of recall bias or the influence of socioeconomic factors. We also did not analyze the presence of any infection signs/symptoms in the caregivers, due to legal regulations, and it is known that RSV transmission is increased in households with symptomatic patients [49]; however, a general rule (which is strictly followed) at our pediatric department is to ask every symptomatic caregiver to be replaced by another person capable of taking care of the child. In spite of its limitations, the study certainly adds to our understanding of the impact of this disease on the caregivers’ QoL. 

## 5. Conclusions

In conclusion, the evidence from this study strongly suggests that caregivers’ QoL is indeed affected by RSV hospitalization. Both the frequency and magnitude of QoL impairment indicate that the problem is not marginal and cannot be neglected. The burden of the RSV goes beyond issues of disease management, and societal losses due to reduced QoL can be observed. Anxiety/depression is the most commonly reported issue among the dimensions assessed by the EQ-5D questionnaire, and although the level of impairment is mild to moderate in the majority of cases, it does reduce caregiver utility. While the descriptive system indicates the QoL domains affected, the reduced utility indicated by the visual analog scale is more likely to reveal more severe QoL impairment, and with regard to the numerical values of utilities, some discrepancies could be observed between the two QoL assessment systems. 

Therefore, we postulate that instruments containing both a descriptive system and a VAS should be used together in studies assessing caregivers’ QoL. The identification of the most affected dimensions could help to introduce counteracting mechanisms and to focus the education of health professionals; this should include information on the psychological aspects of a child’s hospitalization and offer solutions for a more effective patient- and caregiver-oriented approach. This study tentatively points to the problem of RSV-related QoL loss in caregivers of hospitalized children; we argue that once this fact is recognized, problem-oriented countermeasures could be offered and tested in clinical practice. Of note, the loss of utility calculated in caregivers included in this study did not depend on the age of the child or the final diagnosis, suggesting that the fact of hospitalization itself has a significant impact on QoL. Hence, the impact of RSV hospitalization should not be neglected or underestimated when assessing the burden of RSV and its costs, and in the case of economic evaluation of prophylactic or treatment measures, the caregivers’ point of view should also be taken into account. 

## Figures and Tables

**Table 1:** Inclusion and exclusion criteria of caregivers and children in the study.

Inclusion Criteria	Exclusion Criteria
Being a caregiver of an eligible child under 2 years old hospitalized due to RSV infection *	No completion of EQ-5D questionnaire
Being legal guardian of the hospitalized child	Lost to follow-up assessment
Informed consent	Transfer to another hospital
	Not attending the child every day
age under 24 months	
at least one of the signs/symptoms: coryza, cough, increased body temperature or dyspnea	
laboratory confirmation of an RSV infection (with rapid antigen test and/or molecular study)	
community-acquired infection (i.e., signs/symptoms prior to or up to 48 h after hospitalization)	
final diagnosis of pneumonia (J12.1), bronchitis (J20.5), bronchiolitis (J21.0)	

* Inclusion criteria for children to be eligible for the RSV QoL assessment.

**Table 2:** Baseline characteristics of the study group. Abbreviations: Min.—minimum, Max.—maximum, LQ—lower quartile, UQ—upper quartile, *n*—number of patients.

	Median	Min.	Max.	LQ	UQ
whole study group (*n*= 132)					
patients’ male/female ratio (%)	73/59 (55%/45%)				
patients’ age [months]	3.0	0.0	23.0	2.0	6.0
length of stay [days]	7.0	3.0	16.0	6.0	9.0
bronchiolitis (J21.0) (*n* = 100)					
patients’ age [months]	3.0	0.0	19.0	1.0	5.0
length of stay [days]	7.0	3.0	16.0	6.0	9.0
bronchitis (J20.5) (*n* = 10)					
patients’ age [months]	14.5	5.0	23.0	6.0	21.0
length of stay [days]	6.0	4.0	9.0	5.0	8.0
pneumonia (J12.1) (*n* = 22)					
patients’ age [months]	6.0	0.0	23.0	3.0	17.0
length of stay [days]	7.0	4.0	12.0	6.0	9.0

**Table 3:** The baseline caregivers’ quality of life (the kappa coefficient) in the whole study group and with regards to the final clinical diagnosis in hospitalized children—the results from the caregivers’ self-assessment in a disease-free period. * The *p*-value was calculated with the Kruskal–Wallis test with multiple rank comparison. Abbreviations: Min.—minimum, Max.—maximum, LQ—lower quartile, UQ—upper quartile, *n*—number of patients.

	*n*	Median	Min.	Max.	LQ	UQ	*p* *
whole study group	132	1.0	0.9	1.0	1.0	1.0	
bronchiolitis (J21.0)	100	1.0	0.9	1.0	1.0	1.0	0.233
bronchitis (J20.5)	10	1.0	1.0	1.0	1.0	1.0	
pneumonia (J12.1)	22	1.0	1.0	1.0	1.0	1.0	

**Table 4:** The results from the caregivers’ quality of life questionnaire with regard to the assessment systems used (descriptive system and visual analogue scale); all the results were obtained with the EQ-5D-3L questionnaire. Abbreviations: *n*—number of patients, EQ-VAS—EQ-5D-3L visual analog scale, min.—minimum, max.—maximum, LQ—lower quartile, UQ—upper quartile.

	The Study Group (*n* = 132)									
	Descriptive System					EQ VAS				
	Median	Min.	Max.	LQ	UQ	Median	Min.	Max.	LQ	UQ
UTILITY	1.000	0.170	1.000	0.894	1.000	0.860	0.330	1.000	0.800	0.900
UTILITY LOSS	0.000	0.000	0.830	0.000	0.106	0.140	0.000	0.670	0.100	0.200
QALY LOSS	0.000	−2.435	21.904	0.000	1.829	2.451	0.000	16.521	1.370	4.562

**Table 5:** A comparative analysis of the caregivers’ quality of life with regard to the child’s final diagnosis (J21.0—RSV bronchiolitis, J20.5—RSV bronchitis, J12.1—RSV pneumonia); the results were obtained with the EQ-5D-3L questionnaire, the *p* values were calculated with the Kruskal–Wallis test. Abbreviations: *n*—number of patients, EQ-VAS—EQ-5D-3L visual analog scale, LQ—lower quartile, UQ—upper quartile.

		J21.0 (*n* = 100)			J20.5 (*n* = 10)			J12.1 (*n* = 22)			
		Median	LQ	UQ	Median	LQ	UQ	Median	LQ	UQ	*p*
Descriptive system	UTILITY	1.000	0.875	1.000	1.000	1.000	1.000	0.925	0.925	1.000	0.0842
	UTILITY LOSS	0.000	0.000	0.125	0.000	0.000	0.000	0.075	0.000	0.075	0.0842
	QALY LOSS	0.000	0.000	2.170	0.000	0.000	0.000	1.130	0.000	1.849	0.0724
EQ VAS	UTILITY	0.850	0.750	0.900	0.880	0.850	0.900	0.900	0.760	0.970	0.1665
	UTILITY LOSS	0.150	0.100	0.250	0.120	0.100	0.150	0.100	0.030	0.240	0.1665
	QALY LOSS	2.740	1.370	5.137	1.781	1.644	2.466	1.836	0.329	4.603	0.1779

## Data Availability

Data are available upon reasonable request from the authors.
